# Immunomodulatory Mechanism and Potential Therapies for Perinatal Hypoxic-Ischemic Brain Damage

**DOI:** 10.3389/fphar.2020.580428

**Published:** 2020-12-11

**Authors:** Ying-Jun Min, Eng-Ang Ling, Fan Li

**Affiliations:** ^1^ Department of Pathology and Pathophysiology, School of Basic Medical Sciences, Kunming Medical University, Kunming, China; ^2^ Department of Anatomy, Yong Loo Lin School of Medicine, National University of Singapore, Singapore, Singapore

**Keywords:** hypoxic ischemic brain damage, microglia, immune response, immunomodulatory mechanism, therapies, Chinese medicine

## Abstract

Hypoxia-ischemia (HI) is one of the most common causes of death and disability in neonates. Currently, the only available licensed treatment for perinatal HI is hypothermia. However, it alone is not sufficient to prevent the brain injuries and/or neurological dysfunction related to HI. Perinatal HI can activate the immune system and trigger the peripheral and central responses which involve the immune cell activation, increase in production of immune mediators and release of reactive oxygen species. There is mounting evidence indicating that regulation of immune response can effectively rescue the outcomes of brain injury in experimental perinatal HI models such as Rice-Vannucci model of newborn hypoxic-ischemic brain damage (HIBD), local transient cerebral ischemia and reperfusion model, perinatal asphyxia model, and intrauterine hypoxia model. This review summarizes the many studies about immunomodulatory mechanisms and therapies for HI. It highlights the important actions of some widely documented therapeutic agents for effective intervening of HI related brain damage, namely, HIBD, such as EPO, FTY720, Minocycline, Gastrodin, Breviscapine, Milkvetch etc. In this connection, it has been reported that the ameboid microglial cells featured prominently in the perinatal brain represent the key immune cells involved in HIBD. To this end, drugs, chemical agents and herbal compounds which have the properties to suppress microglia activation have recently been extensively explored and identified as potential therapeutic agents or strategies for amelioration of neonatal HIBD.

## Introduction

Neonatal hypoxic-ischemic brain damage (HIBD) is the leading cause of death and long-term neurological deficits in infants and children. It has high incidence (0.3%–0.7%) in neona tes (Chalak, 2012; Hagberg, 2015), with mortality rate (40%) and disability (30%), which brings a heavy burden on families and society (Higgins, 2011; Rocha-Ferreira and Hristova, 2015). Brain injuries in the perinatal period occur in at least four clinical settings: neonatal encephalopathy in term infants, neonatal stroke, encephalopathy of prematurity, and systemic infection, which are related to perinatal hypoxia-ischemia (HI). Ischemia and hypoxia are the main causes of the disease. The most commonly used HIBD models are:Rice-Vannucci model,ischemia-reperfusion model,perinatal asphyxia model and intrauterine hypoxia model. It is well documented that neuroinflammatory response is the key pathogenesis of neonatal HIBD. Central to this is HI which triggers immediate and robust activation of brain immune cells, which include microglia, astrocytes, oligodendrocytes and subsequent infiltration of circulating peripheral leukocytes, lymphocytes etc. Activated brain immune cells along with infiltrated blood cells release a series of inflammatory factors which all together constitute the neuroinflammation after brain injury. Numerous studies have confirmed the important functions of brain resident and peripheral innate immune cells in promoting brain injury and tissue repair in the various stages of the HI (Hagberg, 2012; Bhalala, 2014; Hagberg, 2015). However, the mechanisms of their activation and interaction with brain cells in the immature brain following HI still remain to be fully explored (Li, 2017a). With the recent improvement of obstetrics and neonatal health care, the mortality rate of HIBD has been greatly reduced; yet due to the limitation of treatment, patients who survived HIBD still retain many neurological symptoms or deficits, such as cerebral palsy, epilepsy, and cognitive impairment (Ziemka-Nalecz, 2017; Driscoll, 2018). Currently, most of the treatments for HIBD are based on hypothermia, which requires to be performed within <6 h after birth. HIBD can occur *in utero*, during delivery and after birth. Though MRI can be used to evaluate brain injury in children, the application of this examination would require the full support of anesthesia and other conditions; hence, it has certain limitations especially in the underdeveloped countries and rural areas. Furthermore, it is difficult to use the heart rate detection alone to determine the HIBD (Nelson, 1996). This is compounded by the fact that there are no obvious symptoms in the early stage, thus this time window is often missed (Gunn and Bennet, 2016). In light of this, there is an urgent need to identify more effective treatment strategies targeting at HIBD specifically.

In this review, we will focus on the immune response mechanisms of HIBD especially in neonatal encephalopathy in term infants. To this end, a few potentially effective pharmaceutical therapies for the disease are highlighted.

Four different perinatal animal models are commonly used for study of HIBD pathogenesis. These models aim to simulate hypoxic-ischemic encephalopathy in preterm or full-term infants, but each has its limitations. For example, the classic Rice-Vannucci model has a high mortality rate which is clearly undesirable. The damage of white matter and hippocampus, a hallmark feature of HIBD, in the local transient cerebral ischemia and reperfusion model is not clear. The high animal cost in intrauterine hypoxia model is prohibitive. In the perinatal asphyxia model, it remains uncertain whether it can lead to white matter damage.

## HIBD Animal Models–Advantages and Disadvantages

An appropriate animal model is clearly crucial for studying the pathogenesis of HIBD. The most popular HIBD models adopted are:Rice-Vannucci model,ischemia-reperfusion model,perinatal asphyxia model and intrauterine hypoxia model. However, they all have advantages and limitations. The most ideal model would be the one that share the characteristic features of clinic patients. Neuroinflammation is implicated in different diseases in the nervous system including HIBD, traumatic brain injury and others. Indeed, it is also considered to be one of the core pathogenesis of HIBD. It is well accepted that neuroinflammation can drive the progression of different brain diseases including HIBD as discussed presently in this review. A fuller understanding of neuroinflammation therefore is crucial to development of a proper therapeutic strategy such as the application of different agents or drugs for effective treatment of the disease.

### Classic Rice-Vannucci Model of Newborn HIBD

Most studies have adopted the classic HIBD model i.e., the Rice-Vannucci method (Rice JE, 1981) which mimics neonatal encephalopathy in term infants (Semple, 2013). Briefly, in this method, the common artery of postnatal rats is ligated unilaterally. Following this, the animals are exposed to 8% oxygen at 37°C for 3.5 h. Because of the circulation at the circle of Willis, ligation of unilateral common carotid artery which conceivably would have caused cerebral ischemia may be compensated by contralateral cerebral vessel circulation; hence, it would not result in severe brain damage. However, additional hypoxia exposure after ischemia will cause infarction of the cerebral cortex and hippocampus (Yasuoka, 2004; Stone, 2008; Miura, 2009). This would create a mixed hypoxia and ischemia process which mimics the clinical situations of HIBD in the postnatal rat brain. This model has also been developed in the mice with unilateral carotid ligation +45 min of hypoxia FiO(2) = 0.08 in postnatal day 7 (*p* 7) mice (Stone, 2008) or with unilateral carotid ligation +10% O_2_ in 90% N_2_ for 1 h in postnatal day 10 (*p* 10) mice (Tsuji, 2020).

### Local Transient Cerebral ischemia and Reperfusion Model

This ischemia and reperfusion model was established by Renolleau *et al* (S.Renolleau, 1998). The left middle cerebral artery was permanently electrocoagulated; meanwhile, the left common carotid artery was clamped transiently and then resumed 1 h later at 37°C in seven- day-old Wistar rat pups. This model was aimed to mimic neonatal encephalopathy in term infants (Semple, 2013). Cortical infarction and neuronal apoptosis were observed, although the pups were not subjected to hypoxic exposure. There were no apparent white matter and hippocampus injuries (S.Renolleau, 1998); hence, the experimental model cannot fully mimic the clinical symptoms of HIBD in which lesions in the white matter and hippocampus injuries are hallmark features (Shen, 2012; Zhang, 2013; Zhang, 2020).

### Perinatal Asphyxia Model

This model was established by Bjelke (Bjelke, 1991) through delaying cesarean section which led to intrauterine asphyxia in prenatal rats. The model was designed to mimic neonatal encephalopathy in preterm infants(Semple, 2013). As different from the sheep fetal umbilical cord artery ligation model, pregnant rat uterus surgically removed were readily placed in 37 °C water bath for 14–17 min to cause and mimic different levels of intrauterine pup asphyxia. Hippocampus, cortex, striatum and cerebellum damage was evident in this model (Zhuravin, 2006; Nalivaeva, 2018). There was decrease in cell density and increase in apoptotic cells with mitosis in hippocampal CA1(Daval and Vert, 2004), but white matter damage was not reported.

### Intrauterine Hypoxia Model

Of the different causes of HIBD, maternal HI is one of the most popular pathogenic factors. Ligation of umbilical cord arteries of fetal sheep has been widely used to induce intrauterine hypoxia and ischemia (Gonzalez, 2005; Bertucci, 2011). In this method, the uterine cavity is firstly opened after deep anesthesia of the pregnant sheep. The umbilical artery is completely blocked for 10 min and the circulation is resumed thereafter; this causes fetus intrauterine hypoxia. Although this method replicates the fetal brain damage *in utero*, maternal effects on the fetus cannot be ruled out. It remains uncertain whether the temporary blockage of umbilical cord blood flow alone would have accounted for the observed brain damage. Another point of consideration of this model is severe operational trauma and long recovery time of pregnant sheep. In the latter, the use of big animals may not be cost effective for detailed and meaningful experimental analysis.

All of the above-mentioned four models can simulate HIBD. On the other hand, the first three models have significant advantages over last-mentioned intrauterine hypoxia model in terms of animal selection due to their short breeding cycle and low cost. Both the classic Rice Vannucci and the local transient cerebral ischemia-reperfusion models simulate the hypoxic-ischemic brain injury in term infants. Microglia along with mast cells, monocytes, lymphocytes and neutrophils in the brain injury site are activated. On activation, these brain immune cells secrete a plethora of inflammatory factors which can exacerbate brain injury in term infants after ischemia and hypoxia. Compared with the first two models, the perinatal asphyxia model simulates the hypoxic-ischemic brain damage of preterm infants. Microglia activation and oligodendrocyte precursor cell damage are evident. This is coupled with production of excess amounts of inflammatory factors, oxygen free radicals and glutamic acid, leading ultimately to myelination and synaptic formation disorders (Hagberg, 2015).

## Immune Responses in Neonatal and Adult Ischemic Hypoxic Brain Injury

### Components of the Central immune System

The central nervous system (CNS) is composed of neurons and glial cells comprising microglia, astrocytes and oligodendrocytes. For a long time, due to the existence of the blood-brain barrier, the brain is considered to be an immune-privileged organ (Li, 2017a), but in recent years, many studies have shown that robust immune responses can indeed occur in the brain parenchyma. Additionally, the meninges, cerebral ventricles, and choroid plexus also have the immune responses as in other organs in the body (Shechter and Schwartz, 2013; Louveau, 2015). Recent studies including ours have shown that apart from the normal brain constituent cells, a variable number of immune cells such as lymphocytes exist in the brain parenchyma. In CNS injuries which invariably would result in destruction of the blood-brain barrier and release of chemokines, a large number of peripheral immune cells can enter CNS tissues and elicit an immune response (Liu and McCullough, 2013).

### Differences of Brain immune Response in Neonates and Adults

The immature brain of newborn is different from the mature brain. In the developing brain, the major innate immune cells are microglia, which account for 80% of all immune cells in the cerebrum. Comparing with the adult microglia, microglial cells in the developing brain under normal conditions are more active. They are known as ameboid microglial cells involved in many important activities, such as immune surveillance (Bagheri, 2019), maintaining internal environment stability (Ransohoff and Perry, 2009; Augusto-Oliveira, 2019), and synaptic pruning (Hong, 2016; Matcovitch-Natan, 2016). Ameboid microglia are rapidly activated in brain injuries by assuming a more rounded phenotype, and secrete a variety of proinflammatory- and anti-inflammatory mediators to participate in the immune response. Many studies have reported that HI can induce neuroinflammation in which microglial cells are elicited from the resting form to activated state, and participate in inflammation in the brain. Activated microglia produce and release an array of inflammatory mediators eg interleukin 1β, interleukin 6, and tumor necrosis factor α (Wang, 2013; Li, 2017a); meanwhile, peripheral immune cells (such as mononuclear-macrophages) infiltrated into the brain parenchyma and amplified the brain inflammatory response (Hagberg, 2015). Additionally, astrocytes, as one of the most common glial cells in the brain, also form part of the innate immune response during brain development. They participate in the formation of the blood-brain barrier (Li, 2017a), and are involved in the transport of glutamate in the brain (Meyer, 2017). Moreover, they help transport calcium and potassium ions in the cell (Meyer, 2017), which is related to the oxidative stress process after brain injury. Astrocytes are closely associated with microglia whose interaction can significantly affect the release of inflammatory factors and myelin formation in the brain (Schonberg and McTigue, 2009; Todorich, 2009), thus seriously affect the prognosis of brain injury.

Of note, a small number of myeloid cells are distributed in the brain parenchyma during development, including innate immune cells-monocytes/macrophages, dendritic cells and neutrophils, and adaptive immune cells-T cells, B cells and natural killer cells (Korin, 2017). However, the exact role of these myeloid cells in the developing brain remains obscure. While the roles of these cells during brain development remain uncertain, adaptive immune cells including T cells, B cells and natural killer cells have been found to play a key role in a variety of adult nerve injuries. It has been reported that T lymphocytes can participate in the process of brain injury, and selectively suppress the number of peripheral T lymphocytes can effectively decrease the area of brain tissue damage by reducing the thrombo-inflammatory response to stroke in adults (Kraft, 2013). On the other hand, there is also evidence indicating that suppression of lymphocytes can exacerbate injury in neonatal brain damage (Herz, 2018). This invites speculation of the disparity in immune response of lymphocytes between neonatal and adult brain damages, and that lymphocytes may play different roles during injury process.

In view of the differences in the immune response between the newborn and adult brains, it is suggested that differences might exist in the immune regulation between the newborn and adult brains; hence, the potential treatments for them might also vary. Mortality and high disability make neonatal HIBD one of the main causes of neonatal death and disability, which places a heavy burden on society and families and seriously affects the quality of life of patients. In the present review, we will focus mainly on the immunoregulatory mechanisms of HIBD in the newborns and their potential treatments.

### Regulation of Brain immune Response in Newborns

During brain development, one of the most important physiological activities is synaptic pruning crucial to precise neural circuits in the brain. It has been reported that microglia can participate in synaptic pruning through multiple pathways including complement, TREM2, glutamate transport and inflammation (Min and Li, 2017). There is also evidence that brain immunity can be altered through regulating kv1.2. This is because kv1.2 inhibitor can significantly reduce the concentration of potassium ions in microglia, reduce the production of brain proinflammatory factors such as IL-1β, TNF-α, and reduce the generation of oxygen free radicals (Li, 2008). It has also been reported that Galectin-3, Notch-1, TLR2 and TLR4 can regulate the activation of microglia, thereby affecting the release of proinflammatory and anti-inflammatory factors of microglia, and thus affecting the neuroinflammatory response in the brain (Cao, 2008; Feng, 2011; Li, 2015; Chip, 2017). In our own studies, we have also found that the ATP receptor P2X4 is specifically expressed on the surface of microglia and participates in the regulation of microglia activation. Following administration of P2X4 inhibitors, microglia activation is significantly inhibited which was found to affect not only the neuroinflammatory response but also glutamate clearance of astrocytes and myelin formation in the brain (Li, 2011; Xiao, 2016). This suggests that microglia can affect neuroinflammation in the brain; and in concert with astrocytes, they can affect myelin formation and glutamate clearance in the brain. In addition to the microglia activation after HIBD, the peripheral blood immune system has also been significantly activated. Studies have reported that the increase in peripheral blood leukocytes is positively correlated with the severity of the disease. We have found that peripheral leukocytosis is an early phenomenon after HIBD, and peripheral leukocytes increase is evident within 4 h after HIBD. Coupled with this was the increased expression of various inflammatory reactions (Li, 2016). There is also a significant influx of monocytes/macrophages in the injured brain tissues (Min, 2017).

## Possible Intervention Strategies for HIBD

In consideration of the above, treatment of HIBD may include hyperbaric oxygen chamber, cryotherapy and chemotherapy, and in the latter, both chemical and natural drugs are reviewed and discussed.

### Hyperbaric oxygen chamber and cryotherapy:

It has been reported that hypothermia can significantly reduce the activation of immune system and inhibit the activation of cellular and humoral immunity (Chai, 2005). It can also reduce the infiltration of inflammatory cells in the brain and reduce the release of inflammatory factors (Gu, 2014), thus protecting brain tissue. However, in both instances, the time window is very narrow and limited; treatments need to be started within 6 h after birth when neurological damage symptoms are rarely detected and HIBD diagnosis is hard to make (Jacobs, 2007; Nonato, 2019). In view of this limitation and time constraints, more effective interventions are urgently needed to help improve the prognosis of children with HIBD.

### Chemotherapy

#### Physical Drugs

##### Dehydrating Agent-Mannitol (Antioxidant Stress)

**FIGURE 1:**
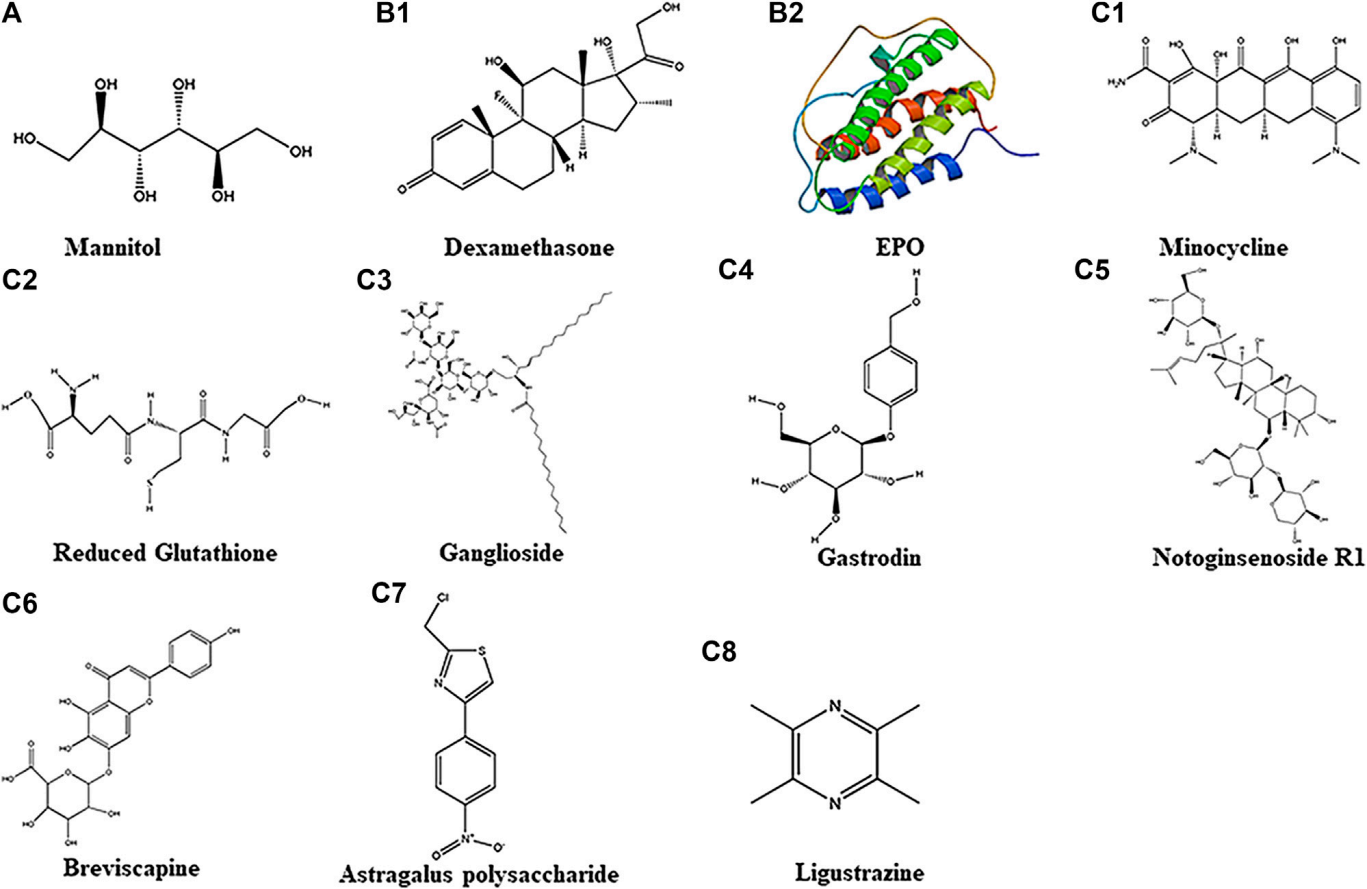
Chemical structure of 11 different drugs. A belongs to Physical Drugs. B1–B2 belong to Drugs With Clear Targets. C1–C8 belong to Chemical Drugs.

During the onset of HIBD, cytogenic brain edema and angiogenic brain edema usually follow; thus, dehydration treatment is routinely used to mitigate the edema. Mannitol is a commonly used drug in clinical practice to reduce intracranial hypertension and improve microcirculation, the chemical structure of which is shown below (Figure 1A). Mannitol is widely used in the treatment of ischemic cerebrovascular diseases such as stroke (Semenenko, 2019) and cerebral infarction (Wang, 2014). Studies have found that in a rat ischemia-reperfusion model, mannitol reduces NO expression and apoptosis in the brain, thereby reducing the damage to cell structure, cerebral infarct size and behavioral deficit after HIBD (Chen, 2018). Because HIBD is followed by cytogenic brain edema and angiogenic brain, dehydration treatment is adopted to mitigate the edema. Studies have reported that mannitol at 2 g/kg in *p* 7 days rat pups could not improve the behavioral scores effectively. However, when the same dose of mannitol was used along with transplantation of HUCB(Human Umbilical Cord Blood, HUCB), the behavioral score of HIBD rats was significantly improved (Yasuhara, 2010). Very interestingly, a small dose of mannitol alone (0.25–0.5 g/kg, intravenous injection) could help recovery of patient'|’s consciousness (Chen and Zhou, 2019) in clinic patients(38–41 weeks), while mannitol at 0.125–1.25 g/kg and HOC (hyperbaric oxygen chamber, HOC) with 0.1Mpa/50 min therapy in combination could improve neurological function score effectively (Liu, 2016). These data supported that mannitol could act as a potential drug to reduce edema, and with its combination with HOC or HUCB transplantation, it could ameliorate HIBD symptoms effectively. On the other hand, its needs to be cautioned that mannitol may cause acute renal failure in the newborns (Liu and Lin, 2001) suggesting the need to routinely and accurately record the urine volume as well as monitor the serum creatinine and urea nitrogen value.

#### Drugs With Clear Targets

##### Glucocorticoids (Antioxidant Stress)

Glucocorticoids, also known as “adrenocortical hormones”, are a class of steroid hormones secreted by the adrenal cortex. It can regulate the biosynthesis and metabolism of sugar, fat, and protein. It also has anti-inflammatory effects and is widely used in clinical practice. As a kind of glucocorticoid, dexamethasone (Figure1B1] at different doses has been shown to possess neuroprotective and neurotoxic effects in brain injury models. This suggests that different doses of dexamethasone may exhibit differences in function in the brain (Lear, 2014). Studies have found improvement in behavioral score of HIBD patients by injecting dexamethasone intraperitoneally 0.5 mg/kg or 0.1 mg/kg at 4 (Ikeda, 2005) or 24 h (Tuor, 1999) before *p* 7 days rats hypoxia (Ikeda, 2005); also, MRI showed improvement in infarct area in which the high-density shadows were reduced under T2 imaging in *p* 7 days rats with Rice-Vannucci HIBD model (Tuor, 1999). In a Rice-Vannucci method developed HIBD model, low-dose (0.1 μg/per animal) dexamethasone intraventricular injection within 2 h after hypoxia could significantly reduce the cerebral infarction area (Harding, 2016). However, by intranasal treatment, even with a high dose (30μg/per animal) of dexamethasone, diminution of ​​cerebral infarction was not apparent (Harding, 2016). Interestingly, some studies confirmed that low-dose dexamethasone given within 24 h after birth (7 days before modeling), and continued administration for three days (0.5 mg/kg on P1, 0.3 mg/kg on P2, 0.1 mg/kg on P3) could aggravate brain damage in an HIBD model (Yeh, 2017). In clinic patients (37–40 weeks), intraventricular injection of dexamethasone at 1 mg/kg within 24 h after birth failed to improve the scores of NBNA. The results suggested that the mode of dexamethasone administration might affect its efficacy. It would appear that for optimal neuroprotective effect, dexamethasone should be administered via intraventricular or intraperitoneal injection within 24 h before HIBD.

##### Erythropoietin (EPO-Promoting Erythropoiesis and Anti-inflammation)

Erythropoietin (EPO) is a glycoprotein hormone which belongs to a colony-stimulating factor. It is synthesized and secreted by liver in infants and kidney in adults. Human erythropoietin has 165 amino acids, and its chemical structural formula is shown below (Figure 1B2). Because EPO is a macromolecular peptide, it is difficult to pass the blood-brain barrier directly. EPO and its receptors are expressed mainly in neurons and glial cells in the hippocampus and cortex (Bernaudin, 1999). It has been reported that neurons and astrocytes can produce endogenous EPO which plays a neuroprotective role in a paracrine manner. In brain damage caused by ischemia, hypoxia, inflammation, trauma, etc. EPO secretion is significantly increased. On binding to its receptor, EPO reduces microglial over-activation and cytokine release, thus exerting its neuroprotective effects (Tamura, 2017). Separately, it has been reported that macrophages with knockout EPO receptors showed weakened phagocytosis, increased apoptotic cells, and induced systemic lupus-like symptoms in mouse. This indicates that EPO plays important roles in macrophages; in other words, the erythropoietin signaling pathway can affect the phagocytic function of macrophages, the innate immune response process, and thus affect the occurrence and development of disease (Luo, 2016). Recent study in a HIBD postnatal rat model had reported that EPO administration effectively reduced the activation of microglia in the brain; of note, the behavioral abnormalities were improved (Lan, 2016). Currently, recombinant human erythropoietin is being used clinically to treat neonatal HIBD; it can increase hemoglobin concentration and improve oxygenation. EPO not only can improve the blood circulation of the newborn and the hypoxic state of the brain to a certain extent, it also possesses neuroprotective functions. It needs to be pointed out that erythropoietin is an endogenous hormone mainly secreted by the liver and kidneys. There is no significant toxic effect when exogenously administered (Xiong, 2011; Yu, 2015). Studies have reported that intraperitoneal injection of 2000–5000 U/kg EPO in the early stage of HIBD rat model (within 24 h after HIBD) could reduce infarct size and incidence of apoptotic neurons, and improve neurobehavioral function simultaneously(Huang, 2019; Xiong, 2019; Yin, 2020). In neonatal MCAO model(*p* 7 days, rat), immediate intraperitoneal injection of 1000 U/kg of EPO after neonatal MCAO could significantly increase its neurogenesis in the subventricular zone and oligodendrocyte generation at *p* 10 days and *p* 21 days(Gonzalez, 2013); however, use of 1000 U/kg of EPO and hypothermia (32°C, 8 h) together after HIBD(established by Rice-Vannucci in *p* 7 days rat) could not improve the behavior scores(Fang, 2013). In HIBD model, EPO at 5000U/kg together with hypothermia (32.5–33 °C, 3 h) or with Huc-MSCs(2×10^6^/ml 0.5 ml) could improve its sensorimotor function significantly(Fan, 2013) and improve behavior score and reduced more injury area than using EPO alone(Yin, 2020). All this suggested that the doses of EPO and the temperature used in hypothermia should to be taken into consideration in order to achieve the best treatment effect for HIBD.

#### Chemical Drugs

##### Minocycline (Anti-inflammation)

Minocycline, also known as dimethylamine tetracycline or cyclamate, is a broad-spectrum tetracycline antibiotic. It has a certain inhibitory effect on Gram-positive bacteria, Gram-negative bacteria, *Mycoplasma*, and *Chlamydia*. The chemical structure is shown below (FIGURE 1C1]. It is fat-soluble and can pass the blood-brain barrier easily. Minocycline can inhibit microglial activation and hyperalgesia in patients with venom-induced persistent spontaneous nociception (Chen, 2013); some studies have confirmed that it may increase the expression of glutamate transporter 1, thereby increasing the clearance of glutamate, reducing the excitotoxicity of glutamate in the brain after HIBD, and exerting neuroprotective effects. Additionally, it could also reduce the inflammation in the neonatal brain by inhibiting neuroinflammation in *p* 0 days rats and P6–7 days rats (Kremlev, 2007; Lechpammer, 2008; Min, 2017), improve myelination in *p* 4 days and *p* 6 days rats (Cai, 2006; Lechpammer, 2008) and reduce brain damage in *p* 0 days rats and *p* 3 days rats (Min, 2017; Wixey, 2018), thereby exerting neuroprotective effects. However, some studies have documented in a model of neonatal reovirus infection that although minocycline can delay the progression of the disease and delay the time when patients develop symptoms of central nervous system infection-viral encephalitis, the high mortality caused by severe encephalitis is ineffective; also, it does not exert significant neuroprotective effects in this disease model (Richardson-Burns and Tyler, 2005).

The efficacy of minocycline related to the age has also be reported. Thus, administration of 40 mg/kg (I.P.) minocycline in *p* 9 days mouse HIBD model could decrease the activation of microglia, but it did not repair long-term behavior abnormalities. However, in a *p* 30 days HI induced brain damage model, the same dose of minocycline, rescued behavioral deficits and inhibited microglia activation significantly (Cikla, 2016). It has been reported that minocycline could suppress microglia activation in a P4d neonatal mouse whereas it did not exert the same effect in the adult mouse brain (Frost, 2019). It has also been reported (Tsuji, 2004) that subcutaneously injected minocycline at 45 mg/kg before HIBD 12 h can exert neuroprotective effects in the rat HIBD model by reducing the injured cells and infarct area, however, intraperitoneally injection of minocycline immediately before HIBD failed to ameliorate brain injury, yet in the mouse HIBD model, subcutaneously injection of minocycline at different doses(45 mg/kg or 135 mg/kg) could aggravate the damage of cerebral cortex, striatum, thalamus, but not hippocampus. It has also been reported that intraperitoneally injection of minocycline with different doses (10 mg/kg or 22.5 mg/kg) can prevent the loss of body weight**,** and reduced the loss of O1-positive and O4-positive cells, as well as microglia activation (Michelle LC, 2008). The disparity in therapeutic effects of minocycline may be attributed to different animal models used; hence, the caution in use of minocycline in clinical setting. Arising from the above, the efficacy of minocycline in brain injury diseases remains controversial. As one of the tetracycline drugs, for newborns with incompletely developed bones, the use of tetracycline drugs might also bring about some undesirable complications, which ultimately would affect the development of the teeth and bones. All this have limited the use of minocycline for the treatment of neonatal ischemic hypoxic brain damage. Because of the significant effect of minocycline in HIBD animals, we expect to see the emergence of more antibiotics with similar structures in the future. It is hoped that they would not lead to undesirable abnormal tooth and bone development in newborn mice. For example, it has been reported that Vancomycin (peptide antibiotics) can reduce the degree of hypoxic-ischemic brain injury in preterm infants by reducing inflammatory response (Lai, 2020), and erythromycin (macrolide antibiotics) can reduce neuronal death ratio after ischemia and hypoxia through antioxidant and anti-inflammatory effects (Lu, 2013; Katayama, 2014). Although these two drugs do not appear to affect the development of teeth and bones, they have potential nephrotoxicity and ototoxicity. There are also reports that ceftriaxone sodium (β - lactam antibiotics, cephalosporins) can reduce the number of apoptotic neurons in hippocampus and improve learning and memory impairment (Lai, 2011), However, it has potential hepatorenal toxicity.

##### Adenosine triphosphate Receptor Antagonist

Adenosine triphosphate ATP has long been considered as a substance that can store energy and provide the energy needed by the body. ATP receptors are divided into adenosine receptor (P1) and purine receptor (P2). The adenosine receptors can be divided into four types: A1, A2A, A2B, and A4. Purine receptors can be divided into two types: ionic P2X receptors and metabolic P2Y receptors. Currently, seven ionic P2X receptors and 13 metabolic P2Y receptors have been identified. When neurons are excited, they can release ATP and act on other nerve cells. ATP plays an important role in the transmission of neural excitation signals. We have reported that the ATP receptor P2X4 is expressed in the postnatal rat brain within the first 14 days after birth (Li, 2011). P2X4 expression is markedly increased after brain injury in neonatal rats induced by hypoxia alone. Furthermore, it is specifically blocked by the combination of blockers ATP-TNP and PPADS. Remarkably, the long-term behavioral abnormalities induced by systemic hypoxia were improved; also, the 7 T magnetic resonance results revealed that the abnormal formation of myelin sheath caused by hypoxia can be repaired (Xiao, 2016).

Studies have found that purine receptor and adenosine receptor inhibitors can also affect energy metabolism and, very interestingly, the functions of microglia, thereby affecting the innate immune response and exerting brain protective effects. Studies have also confirmed that P2X7 receptors are widely expressed in the cerebral cortex and hippocampus of human and rodent (He, 2012). They can mediate microglial activation and affect the inflammatory response as well as synaptic transmissions in the brain. P2X7gene knockout will downregulate glutamate concentration, resulting in impaired neurotransmission in hippocampus (Papp, 2004). Studies have also confirmed that P2X7 receptors can mediate apoptosis and autophagy by activating cysteine proteases. Under the action of various pathogenic factors (oxidative stress, inflammation, trauma or hypoxia ischemia, etc.), the P2X7 receptor can exert its effects by regulating the intracellular Ca^2+^ concentration, producing and releasing IL-1β and glutamic acid and play protective effects. Metabolic purine receptor P2Y12 inhibitor found in ischemic cardio-cerebral vascular disease can significantly inhibit the expression of ATP hydrolysate adenosine 5′-diphosphate, thereby inhibiting platelet aggregation, reducing incidence of myocardial infarction, stroke, severe recurrence ischemia in patients, transient ischemic attack, arterial thrombosis. P2Y12 has also been confirmed to be widely expressed in mouse microglia. After knocking out P2Y12, the microglia phenotype remained unaffected; however, microglial migration and activation were blocked. After administration of P2Y12 inhibitors, it was also found that microglia activities and release of pro-inflammatory factors were decreased (Haynes, 2006).

##### White Blood Cell Inhibitors (Anti-inflammation)

Recent studies have reported in an adult stroke model that FTY720 extracted from Chinese herbal *Cordyceps sinensis* can inhibit the peripheral blood lymphocytes (Norimatsu, 2012). In this connection, it is relevant to note that MC21, an antibody which can block the expression of CCR2 specific in myeloid cells, can inhibit myeloid cells function significantly, especially by inhibiting the function of monocytes (Mack, 2001), thereby reducing the inflammatory response, reducing the adaptive immune response in the brain, and improving the prognosis of patients. Contrary to the above, it has also been reported that in a permanent electrocoagulation of the right common carotid artery mouse model of ischemic hypoxic brain damage that FTY720 at 1 mg/kg and given by intraperitoneal injection within 20 min after HIBD to inhibit lymphocytes may aggravate brain injury in *p* 9 days mouse (Herz, 2018). This suggests that the actions of lymphocyte inhibitors in neonatal and adult diseases may be inconsistent. However, in a newborn rat HIBD model by Rice-Vannucci method and lipopolysaccharide, after using FTY720 at 0.3 mg/kg by intraperitoneally injection within 30 min after HIBD to eliminate lymphocytes, the results showed an obvious reduction of neuroinflammatory reaction, and the incidence of cerebral palsy was decreased coupled with recovery of the motor function (Yang, 2014). These two completely conflicting results may be attributed to different animal models used. Immune response is distinctly different in adult and neonatal brain injury model. In adult mouse brain damage, in addition to innate immune cells - microglia activation, adaptive immune cells: T and B lymphocytes infiltrate into brain and release inflammatory factors, and then participate in the process of brain injury. In neonatal brain injury, the inflammatory response in the brain mainly involve microglial cells, monocytes, dendritic cells, etc., in the acute phase of cerebral injury (within 2 weeks after injury). It is to be noted that T lymphocytes do not infiltrate into the damaged parenchymal (Li, 2017a). Therefore, the need to carefully evaluate the experimental animal model before application of FTY720.

##### Calcium Channel Antagonists

In the onset of clinical HIBD children, ischemia is often accompanied by hypoxia. Nimodipine has been reported to specifically expand the cerebral blood vessels and increase the coronary blood flow. While lowering the blood pressure, it guarantees the intracranial blood supply and has a protective effect on ischemic hypoxic brain damage (Qu, 2004). Flunarizine, as a fourth-generation calcium antagonist commonly used in clinical cardiology, can relax the vascular smooth muscle, reduce blood pressure, reduce myocardial oxygen consumption, and increase the ability to resist hypoxia.

##### Antiplatelet Aggregation Drugs

The change of platelet morphology and function is a major influential factor in ischemic diseases. The main role of antiplatelet drugs is to prevent platelet aggregation and adhesion, which can effectively prevent thrombosis. At present, antiplatelet aggregation drugs are mainly divided into the following four according to different blocking pathways: a. Cyclooxygenase inhibitors, such as aspirin, b. adenosine diphosphate receptor blockers, such as P2Y12 receptor inhibitors-clopidogrel, c. Phosphodiesterase inhibitors, including dipyridamole and cilostazol, and d. glycoprotein IIb/IIIa receptor blockers, such as tirofiban, etebase, acyximab. Studies have reported that long-term low-dose aspirin can significantly reduce the incidence of stroke, but once discontinued, it will increase the risk of stroke (Rodriguez, 2011). Clopidogrel, as an inhibitor of the P2Y12 receptor, can inhibit platelet activity and reduce thrombosis through the metabolic production of CYP450 enzymes, thereby exerting its neuroprotective effects.

##### Oxygen Free Radical Scavenger (Antioxidant Stress)

Oxidative stress is produced when reactive oxygen species production exceeds that of endogenous antioxidant system. Reduced glutathione is the most important antioxidant, the chemical structure of which is shown below (Figure 1C2). It contains 90% non-protein thiols in the cell and 85% non-protein thiols in the plasma, and together with oxidized glutathione, they constitute the most important redox buffer pair in the body (Reid and Jahoor, 2001). As a low-molecular free radical scavenger, reduced glutathione markedly increases the activity of oxidase, thereby oxidatively decomposing the superoxide groups formed due to ischemia, hypoxia, trauma etc., and inhibiting formation of lipid peroxides, thereby inhibiting tissue damage from oxygen free radicals. Clinical studies have shown that reduced glutathione effectively elevates oxidase activity and inhibits the formation of oxygen free radicals, thereby exerting brain protection function. Concurrently, the combined use of reduced glutathione (300 mg) and gangliosides (20 mg) for 7 days (mild injury) or 14 days (moderate injury) can activate ATPase on the brain cell membranes after brain tissue damage. Along with this, neuronal cell edema can be reduced and its damage is further attenuated. This is coupled with return of consciousness and muscle tension, and disappearance of convulsions (Wei, 2019). Also, it can effectively increase oxidase activity and improve the scores of NBNA and CT value of low-density lesion in the white matter after HIBD (Wang and Li, 2018).

##### Cell Membrane Stabilizer (Stable Cell Membrane)

Ganglioside is a glycosphingolipid, which is one of the constituents of animal cell membranes. It is abundant in the nervous system; the chemical structure is shown below [Figre 1C3]. After brain injury, gangliosides, especially monosialic acid gangliosides, can cross the blood-brain barrier under hypoxic conditions, inhibit NO_2_ synthesis, and stabilize neuronal cell membranes (Wang, 2019). They can improve brain edema (Yang and Zhang, 2020), correct intra- and extra-cellular ion imbalance, neural function and behavior recovery, and reduce mortality. Meta-analysis shows that ganglioside as an adjuvant therapy can significantly alleviate neurodevelopmental disorder, cerebral palsy, intellectual disability etc. It has a remarkable effect on improving the neurobehavioral function of HIBD (Sheng and Li, 2017). Clinical studies also showed that the combined use of gangliosides and EPO can reduce the mortality and the incidence of cerebral palsy in children with HIBD; furthermore, the combination can also relieve the muscle tension and improve the neurological deficit (Zhu, 2015). The combined use of ganglioside(20 mg) and hypothermia(33–34.5°C) can improve the scores of NBNA, MDI and PDI (Zhou, 2020). Also, combined use of ganglioside(20 mg) and HOC(0.03–0.04Mpa/30 min) can improve the scores of NBNA and reduce inflammation(Zhang, 2018).

##### Stem Cell Therapy

Among the neurological sequalae of HIBD, such as cerebral palsy, cognitive decline, and mental symptoms, neuronal death is featured prominently. As non-renewable cells, neurons affected by adverse conditions are incapable of repairing the brain damage through self-proliferation. Therefore, many studies have attempted to promote the regeneration of neurons and repair of neural tissues in the hope that some neurological functions may be restored. The main aim is to reduce the occurrence of sequelae in children with HIBD. In recent years, stem cell transplantation has become a focus of many studies in neuro-generative medicine, and some promising results have been gained in the treatment of cerebral ischemic diseases (Xie, 2016). Stem cells refer to cells that have multidirectional differentiation and self-renewal ability and can differentiate into cells similar in structure and function to the surrounding cells under the induction of their ambient microenvironment. Previous studies have confirmed that mesenchymal stem cells can differentiate into neurons and glial cells under certain induction conditions. Studies have shown that the use of embryonic stem cells for the treatment of HIBD model in mice has significantly improved the learning and memory abilities, as well as limb movement capabilities (Ma, 2007; Shinoyama, 2013; Hawkins, 2018). In 2015, a study combining mild hypothermia therapy with human cord blood-derived mesenchymal stem cell transplantation was used to treat HIBD in rats. It was found that combined therapy can increase the population of astrocytes in the mouse brain and improve the sensory and motor function (Park, 2015).

##### Gastrodin (Antagonizing Glutamate Excitotoxicity, Anti-inflammation)

Gastrodin, the main component of *Gastrodia,* is chemically named as 4-hydroxymethylphenyl-β-d-glucopyranoside, and whose chemical structure is shown in (Figure 1C4). As a sedative, Gastrodin exerts calm and analgesic effects. It improves the modulatory ability of cerebrovascular significantly and has obvious anti-inflammatory and antioxidant function. Recent studies have shown that it can increase vascular compliance and has a good therapeutic effect on CNS diseases caused by ischemia (Zhu, 2012; Li and Zhang, 2015; Shi, 2019).

Studies have found that in drug-induced epilepsy models, Gastrodin can reduce the number of NMDA receptors in hippocampus, thereby minimizing neural excitability toxic and neuroprotective effects (Wong, 2016). It can also reduce brain infarct and edema and improve neural function in a rat middle cerebral artery embolism model. By using cultured hippocampal primary neurons glucose and oxygen deprivation model, it was reported that Gastrodin at different doses (100 μg/ml,200 μg/ml,13 mg/L,26 mg/L, 52 mg/L) added in the medium can reduce neuronal apoptosis, decrease intracellular calcium ions and extracellular glutamate concentration and prevent neurotoxicity (Mao, 2007; Xu, 2007). In a HIBD model, it was also found that Gastrodin can inhibit the activation of microglial cells, reduce the release of inflammatory factors and incidence of apoptotic neurons through the renin angiotensin system and Sirtuin3 pathway (Liu, 2018; Guo, 2020). Also, Gastrodin at 1000 mg/kg administered by intraperitoneal injection after HIBD in *p* 7 days rats can decrease prostaglandin and thrombosis (Niu and Jin, 2008).

##### Notoginsenoside R1(Antioxidant Stress, Anti-inflammation)

Notoginsenoside R1 is one of the main components of Notoginsenosides extracted from the rhizome of Notoginseng. Its molecular formula is C47H80O18 and its chemical structure is shown below (Figure1C5). It has the functions of dispersing blood stasis, hemostasis, swelling, analgesia, anti-inflammatory and anti-oxidant (Liu, 2010). Notoginsenoside R1 can promote microcirculation and reduce blood stasis. It can reduce platelet activity, inhibit platelet aggregation and adhesion, and reduce thrombosis, thereby significantly improve microcirculation and metabolic disorder in damaged neurons caused by ischemia and hypoxia. As an extract of *Panax notoginseng,* it also has an estrogen-like effect, also known as natural phytoestrogens (Chan, 2002), and can exert anti-inflammatory, antioxidant, anti-apoptotic effects through estrogen receptors; and under hypoxia it renders a neuroprotective role in ischemic encephalopathy (Tu, 2016).

In a study using the rat hippocampal neuron culture subjected to glucose and oxygen deprivation and re-enrichment *in vitro*, it was reported that Notoginsenosides could reduce the formation of oxygen free radicals, reduce intracellular calcium overload, thereby reducing the hypoxia and ischemia resulted injury (Zhu, 2003). *Panax Notoginsenoside* R1 is an estrogen-like substance that can stimulate estrogen receptors, activate the AKT signaling pathway, inhibit endoplasmic reticulum stress, and exert neuroprotective effects (Meng, 2014). However, the neuroprotective effect of Notoginsenoside R1 was significantly inhibited after administration of estrogen receptor blockers (Tu, 2016), lending to the support that Notoginsenoside R1 acts through the estrogen receptor.

It was reported that Notoginsenoside R1 at different doses(10 mg/kg and 15 mg/kg) can reduce infarction volume post-injury and cell death in *p* 7 days rats after HIBD, while 5 mg/kg Notoginsenoside R1 failed to reduce infarction volume post-injury(Wang, 2016). Additionally, in the primary rat neuron culture, only 5 μmol/L and 10 μmol/L Notoginsenoside R1 can attenuate cell apoptosis, 0.5–2 μmol/L Notoginsenoside R1 all failed to attenuate cell apoptosis(Wang, 2016; Tu, 2018). While in HIBD rat model, intraperitoneally injection of Notoginsenoside R1 at 15 mg/kg can reduce infarction volume, and improve neurobehavioral function.

##### Breviscapine (Anti-platelet Aggregation, Anti-inflammation)

Breviscapine*,* a member of the *Asteraceae* family, is an active ingredient extracted from the endemic plant of Breviscapus in Yunnan, China. Its chemical formula is C42H36O23 whose chemical structure is shown below (Figure 1C6]. The main component is baicalein, which can expand the cerebral blood vessels then increase the cerebral blood flow. It prevents coagulation by resisting platelet aggregation and reducing plasma viscosity. It is widely used in prevention and treatment of ischemic hypoxic related cardiovascular as well as cerebrovascular diseases clinically.

It is documented that Baicalein the main component of Breviscapine can reduce the intracellular calcium overload by inhibiting protein kinase and has anti-oxidative stress function thereby protecting the brain from ischemia and hypoxia injury (Li, 2017b). In a separate study, it was found that Breviscapine could improve Bcl-2 expression and inhibit Bax expression in a neonatal rat HIBD model (Zhang, 2011), concomitantly, neuronal apoptosis was decreased. In clinic study, it was found that intravenous injection of Breviscapine(4 mg) can increase SOD production but decrease MDA level in serum. It can promote muscle tone recovery and reduce inflammation (Wang, 2011; Li, 2014; Zhang and Xiao, 2017). Combined use of Breviscapine (4 mg) and ganglioside (20 mg) or HOC (0.04–0.06Mpa/60 min) can improve the scores of MDI, PDI, NBNA and neurological function(Li, 2013; La, 2018).

##### Astragalus Membranaceus (Improving Immune Response)

Astragalus is extracted from the root of Mongolian legume Astragalus. Astragalus contains Polysaccharides, Saponins, Isoflavones, Flavonoids etc. Astragalus polysaccharide is the main active ingredient. The chemical structure of Astragalus polysaccharide is shown below (Figure 1C7.

Some studies support that Astragalus improves the immune function of the human body significantly. Astragalus polysaccharide has been shown to increase the expression of cell surface adhesion molecules, and promote the adhesion of vascular endothelial cells and lymphocytes which therefore accelerate the circulation of lymphocytes resulting in immunity activation. Other studies have found that, Astragalus can reduce brain excitatory amino acid concentration (Wang, 2006), enhance mitochondria scavenge oxygen free radicals, thus reducing the production of oxygen free radicals and lipid peroxides, resulting in reduced apoptosis *in vitro*. Through this process, it protects the brain from injury (Min, 2005; Li, 2019). It has been reported that intravenous injection of Astragalus polysaccharide can reduce the incidence of cerebral palsy, prevent intellectual development lag (Zhuo, 2009), relieve immune dysfunction (Zhang and Zhao, 2013), improve the scores of NBNA, and reduce inflammation (Yu and Zhang, 2018; Li and Yu, 2019).

##### Ligustrazine (Reducing Calcium Ion Concentration)

Ligustrazine is an alkaloid extracted from the plant Ligusticum whose chemical structure is shown below (Figure 1C8). Due to its effect of preventing platelet aggregation, dilating small arteries, improving microcirculation and cerebral blood flow, it is widely used in clinical vascular disorders, such as occlusive vascular disease, cerebral thrombosis, vasculitis, treatment of coronary heart disease, angina pectoris among others.

Studies have reported that Ligustrazine can reduce intracellular calcium concentration, inhibit proliferation of vascular smooth muscle cells and increase blood flow, as well as play a protective role in ischemic diseases. Ligustrazine also affects the number of peripheral blood neutrophils, monocytes/macrophages and lymphocytes, and regulates the innate immune response and adaptive immune response, thereby enhancing the immune function of human body. Li et al. reported that, Ligustrazine can reduce apoptotic neuronal number in an adult bilateral common carotid arteries ligated hypoxia and ischemia brain injury rat model, and in neuronal cell line PC12 ischemia and hypoxia stimulation model by glucose and oxygen deprivation (Zhao, 2018). In clinic study, it was found that Ligustrazine alone at different doses (2–4 mg/kg or 6–8 mg/kg) can improve the scores of NBNA and improve neurological function (Zhang and Cao, 2008; Zhang, 2009; Zhao, 2011; Jiang, 2014; Qiu, 2014).

Apart from the above, a large number of hormones and Chinese herbal agents have been reported to possess neuroprotective effects. For example, Melatonin can play a neuroprotective role through anti-oxidative stress, anti-inflammatory, inhibition of cell autophagy and programmed cell death (Aly, 2015). Vitamin D can exert neuroprotective effects through antioxidant stress (Zhang, 2011). Salvia miltiorrhiza is a clinically effective drug that accelerates blood circulation. It also reduces ischemia-reperfusion infarction significantly by diminishing the number of white blood cells, especially neutrophils, thereby reducing the inflammation (Liu, 1998; Liu, 1999). Resveratrol removes oxygen free radicals, anti-oxidative stress, and reduces the area of cerebral infarction after HIBD(Le, 2019). Traditional Chinese medicine Geniposide activates the PI3K/Akt signaling pathway to reduce cytokine production in HIBD(Liu, 2019). Cannabidiol is one of the natural ingredients extracted from Cannabis plant; it improves the myelin formation related disorders caused by ischemia and hypoxia in newborn rats which ultimately improves the survival of neurons and reduces the number of microglial cells (Ceprian, 2019). Ginkgo biloba extract was found to regulate the expression of neuron-specific enolase and S-100 protein in the brain, thereby improving intracellular metabolism, reducing calcium overload, and exerting neuroprotection functions (Hou, 2003). It has also been reported that *Lycium barbarum* polysaccharide prevents the damages of neuron cell membrane through antioxidant stress (Gong, 2009; Ma, 2018), and curcumin can protect against HI diseases through anti-inflammatory and antioxidant stress effects (Wang and Zhu, 2013).

**FIGURE 2:**
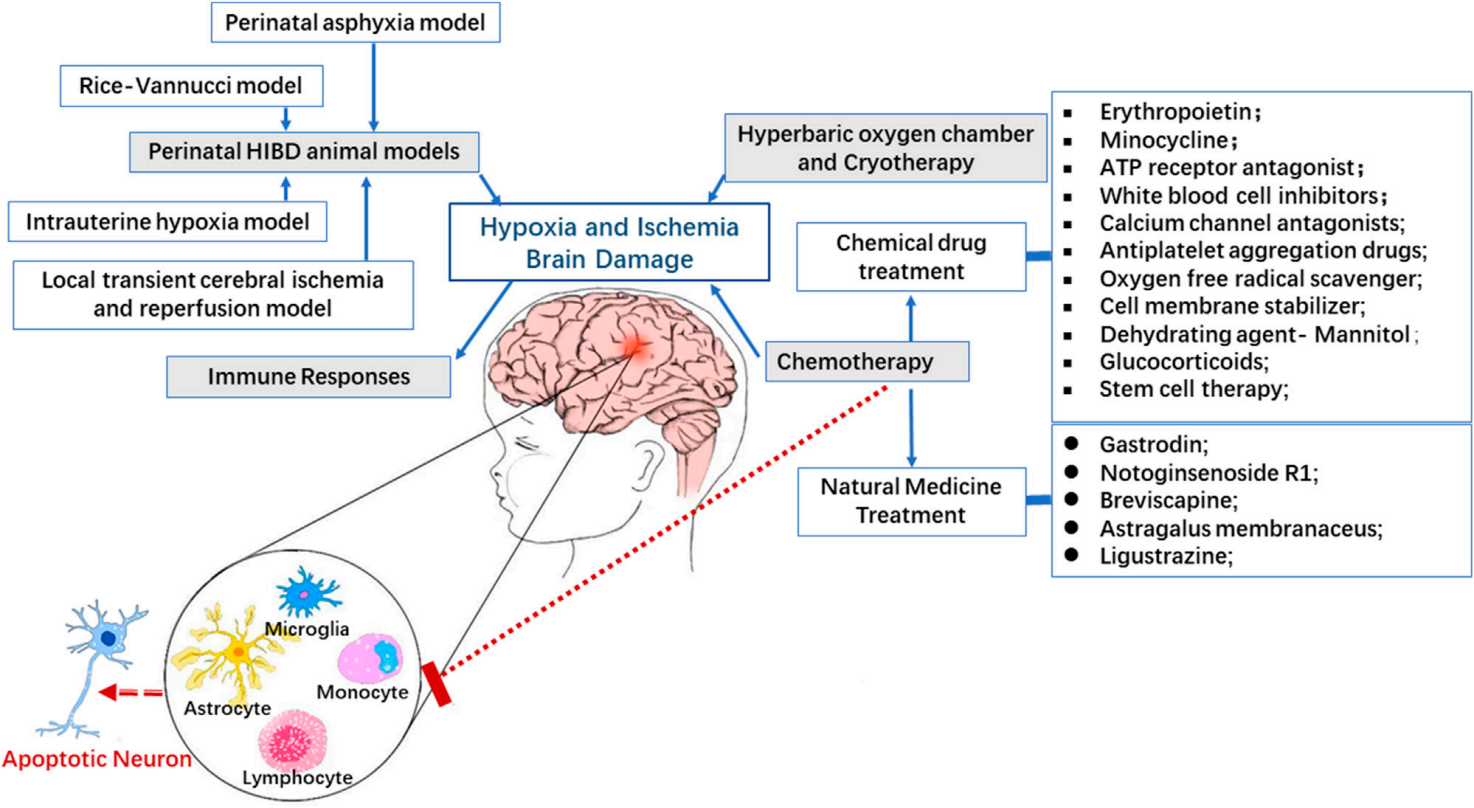
Immunomodulatory mechanism and potential therapies in HIBD. Active immune response plays a key role in the pathogenesis of perinatal HI brain injury. There is compelling evidence indicating that regulation of immune response involving microglia, monocytes and lymphocytes can effectively rescue the outcomes of brain injury in which apoptosis of neurons is featured prominently in experimental perinatal hypoxic ischemic model.

The immune response or activation is one of the core pathogenesis of HIBD. In the present review, three main types of drugs, namely, physical drugs, drugs with clear therapy target, and drugs without clear therapy target, have been discussed in connection with their properties that can suppress the immune response as well as repair brain damage in some clinical trials or/and animal models by different mechanisms. The logical relationship of HIBD, immune response and intervention is shown in Figure 2. All evidences seem to indicate and converge that immune response modulation especially regulation of microglial activation should be a potential therapy target of HIBD.

## Conclusion

Because of the destructive long-term neurological outcomes and limited treatments, it is crucial to decipher the exact mechanisms underlying HIBD and explore for more effective treatment of the disease. Immune response is deemed to be one of the core pathogenesis of HIBD; thus, focusing on this process should provide the possibility to develop safer and more effective treatments for HIBD. In the past few years, accumulating evidence derived from many studies using different experimental animal and cell models have shown that certain anti-inflammatory agents along with some natural Chinese compounds including those mentioned above, can effectively alleviate brain tissue damage caused by ischemia and hypoxia. Currently, EPO, reduced glutathione, Ganglioside, Mannitol, Breviscapine, Astragalus polysaccharides and Ligustrazine have been used in the clinical treatment of HIBD. FTY720 has also been used to suppress immune rejection after renal transplantation and has entered a phase III clinical trial; it is a very promising drug for clinical treatment in the future. Although minocycline is used in adult diseases, it affects the normal development of bones in children, which limits its usage in clinical treatment. Nonetheless, based on the progress of the basic research of minocycline, in the future, there may be available structurally similar alternative drugs that do not affect bone development for the clinical treatment of HIBD. It would appear that the different compounds or agents as discussed in this review can act through different pathways and play an essential role in neuroprotection. The list of these compounds or agents including their properties, application and therapeutic effects in different animal models are summarized in Table 1.

**TABLE 1:** Pharmacological properties of different drugs.

Drug name	Mechanisms of action	Effects	Medication requirements	Advantages	Disadvantages
Mannitol	Reduce brain edema	Antioxidant stress	Intracranial active bleeding is disabled, acute pulmonary edema and severe water loss patients is disabled, severe pulmonary congestion and acute tubular necrosis patients without urine is contraindicated	Early administration	May impair kidney function
Dexamethasone	Reduce the extent of cerebral infarction	Antioxidant stress	Be allergic to this drug and adrenal cortex hormones are prohibited	Early and preventive administration	Affect blood cortisol concentration
EPO	Improve oxygenation	Promoting erythropoiesis and anti-inflammation	Disabled in patients with severe hypertension and who are allergic to human serum albumin and those who are coinfected	Safe and convenient	Overdose can cause cardiovascular system complications
Minocycline	Reduce inflammation	Anti-inflammation	Prohibited for those allergic to this drug and tetracycline drugs	Significantly inhibits inflammation	Affects tooth and bone development
Reduced- glutathione	Reduce cell edema	Antioxidant stress	Use with caution for those allergic to this medicine	Easy to administer	Used alone, the effect is not good
Ganglioside	Improve neurobehavior	Stable cell membrane	Use with caution for those allergic to this medicine, disabled for inherited abnormal glucose and lipid metabolism and Guillain-Barré syndrome	Easy to administer	Used alone, the effect is not good
Gastrodin	Improve nerve function	Antagonizing glutamate excitotoxicity	Prohibited for those allergic to this drug	Safe and low toxicity	The ingredients are not single
Notoginsenoside R1	Reduce nerve cell damage	Antioxidant stress	Uncertain	Safe and effective	The ingredients are not single
Breviscapine	Protect nerve cells	Anti-platelet aggregation	Uncertain	Protects organs from adverse reactions	The ingredients are not single
Astragalus- polysacchari	Protect nerve cells	Improving immune response	Uncertain	Safe and effective	The ingredients are not single
Ligustrazine	Neuroprotective effect	Reducing calcium ion concentration	For those who are allergic to this drug and patients with cerebral hemorrhage	Safe and effective	The ingredients are not single
FTY720	Reduce inflammation	Anti-inflammation	Uncertain	Significantly inhibits inflammation	Affect the number of granulocytes

## Author Contributions

Y-JM prepared the first draft of the manuscript. The study was conceptualized by FL and E-AL who also supervised the work and reviewed the entire manuscript.

## Funding

This work is supported by the National Natural Science Foundation of China (NO. 81760280), and Yunnan Natural Science Foundation of China (NO. 2018FE001 (-002) to FL.

## Conflict of Interest

The authors declare that the research was conducted in the absence of any commercial or financial relationships that could be construed as a potential conflict of interest.

## Glossary

ATP: Adenosine 5′-triphosphate
ATP-TNP: 2′, 3′-0-(2, 4, 6-Trinitrophenyl) adenosine 5′-triphosphate
BCAO: Bilateral Carotid Artery Occlusion
CCL: Unilateral ligation of the common carotid artery
CCR2: C-C chemokine receptor type 2
CNS: Central nervous system
CYP450: Cytochrome P450 monooxygenase
EPO: Erythropoietin
FTY720: Fingolimod hydrochloride
HI: Hypoxia-Ischemia
HIBD: Hypoxic-ischemic brain damage
h: hour
HOC: Hyperbaric Oxygen Chamber
hs-CRP: High Sensitivity C-Reactive Protein
HUCB: Human Umbilical Cord Blood
hUC-MSCs: human Umbilical Cord Mesenchymal Stem Cells
IL-1β: Interleukin 1β
I.C.V.: Intracerebroventricular Injection
I.P.: Intraperitoneal Injection
I.V.: Intravenous Injection
LPO: Lipoperoxides
MCAO: Middle Cerebral Artery Occlusion
MDA: Malondialdehyde
MDI: Metal Development Index
NBNA: Neonatal Behavioral Neurological Assessment
NO2: Nitrogen dioxide
NO: Nitric oxide
OGD: Oxygen-Glucose Deprivation
PDI: Psychomotor Development Index
PPADS: Pyridoxal phosphate-6-azo (benzene-2, 4-disulfonic acid)
PTC: Plasma Total Cortisol
S.C.: Subcutaneously Injection
sICAM-1: soluble Intercellular Adhesion Molecule-1
SOD: Superoxide dismutase
TNF-α: Tumor Necrosis Factor-α
TREM2: Triggering receptor expressed on myeloid cells 2
TLR2: Toll like receptor 2
TLR4: Toll like receptor 4
